# Critical requirement of SOS1 RAS-GEF function for mitochondrial dynamics, metabolism, and redox homeostasis

**DOI:** 10.1038/s41388-021-01886-3

**Published:** 2021-06-12

**Authors:** Rósula García-Navas, Pilar Liceras-Boillos, Carmela Gómez, Fernando C. Baltanás, Nuria Calzada, Cristina Nuevo-Tapioles, José M. Cuezva, Eugenio Santos

**Affiliations:** 1 Centro de Investigación del Cáncer-Instituto de Biología Molecular y Celular del Cáncer (CSIC – Universidad de Salamanca), Salamanca, Spain; 2 Centro de Investigación Biomédica en Red de Cáncer – Cáncer (CIBERONC), Madrid, Spain; 3 Departamento de Biología Molecular, Centro de Biología Molecular Severo Ochoa3, (CSIC – Universidad Autónoma de Madrid), Madrid, Spain; 4 Centro de Investigación Biomédica en Red de Cáncer – Enfermedades Raras (CIBERER), Madrid, Spain

**Keywords:** Oncogenes, Cell signalling, Molecular biology

## Abstract

SOS1 ablation causes specific defective phenotypes in MEFs including increased levels of intracellular ROS. We showed that the mitochondria-targeted antioxidant MitoTEMPO restores normal endogenous ROS levels, suggesting predominant involvement of mitochondria in generation of this defective SOS1-dependent phenotype. The absence of SOS1 caused specific alterations of mitochondrial shape, mass, and dynamics accompanied by higher percentage of dysfunctional mitochondria and lower rates of electron transport in comparison to WT or SOS2-KO counterparts. SOS1-deficient MEFs also exhibited specific alterations of respiratory complexes and their assembly into mitochondrial supercomplexes and consistently reduced rates of respiration, glycolysis, and ATP production, together with distinctive patterns of substrate preference for oxidative energy metabolism and dependence on glucose for survival. RASless cells showed defective respiratory/metabolic phenotypes reminiscent of those of SOS1-deficient MEFs, suggesting that the mitochondrial defects of these cells are mechanistically linked to the absence of SOS1-GEF activity on cellular RAS targets. Our observations provide a direct mechanistic link between SOS1 and control of cellular oxidative stress and suggest that SOS1-mediated RAS activation is required for correct mitochondrial dynamics and function.

## Introduction

SOS1 and SOS2 are the most universal and widely expressed RAS-GEFs (guanine nucleotide exchange factors) in metazoan cells [1–7]. Despite their similar protein structures and expression patterns, most studies analyzing genetically modified mouse models support a dominant in vivo functionality of SOS1 over SOS2 in different biological contexts [8–12]. Specifically, phenotypic and functional studies of primary mouse embryonic fibroblasts (MEFs) derived from SOS1-KO, SOS2-KO, and SOS1/2-DKO mice have demonstrated functional prevalence of SOS1 over SOS2 in control of a wide variety of pathological and physiological cellular processes. Thus, a critical role was demonstrated for SOS1 in development of BCR-ABL-driven leukaemia as well as in skin homeostasis and chemically induced carcinogenesis [13–16]. Functional prevalence of SOS1 over SOS2 was also reported in physiological processes including control of cell proliferation and viability, modulation of migratory and inflammatory cellular processes, or regulation of intracellular ROS levels [12, 17, 18].

Regarding the functional specificity/redundancy of SOS1, it is relevant to understand the molecular mechanisms underlying the markedly increased levels of ROS and oxidative stress that are specifically detected in MEFs devoid of SOS1 [7, 17]. In particular we wished to get functional insights into the mechanistic details mediating the altered redox phenotype, as well as conclusively identifying the source of the elevated ROS in SOS1-deficient fibroblasts. To this end, here we performed detailed analyses of mitochondrial morphology and function in MEFs of relevant SOS genotypes (WT, SOS1-KO, SOS2-KO, SOS1/2-DKO) and also carried out functional and metabolic profiling of these SOS-devoid cells. The mitochondrial and metabolic profiles of SOSless MEFs have also been compared to those of RASless MEFs [19, 20] with an aim at pinpointing potential mechanistic contribution of RAS proteins to generation of the phenotypes of SOSless MEFs. Our observations support a specific mechanistic link between SOS1 and control of intracellular redox homeostasis and mitochondrial function, and suggest that activation of RAS proteins by SOS1 is a critical requirement for maintenance of correct mitochondrial structure, function, and respiratory/metabolic homeostasis.

## Results

### Increased oxidative stress of SOS1-deficient MEFs is reversed by mitochondria-targeted antioxidant MitoTEMPO

We reported previously that SOS1 depletion (but not SOS2 depletion) causes specific phenotypic defects in primary MEFs, including in particular a substantial increase of intracellular ROS and oxidative stress [12, 17, 18].

**Fig. 1:**
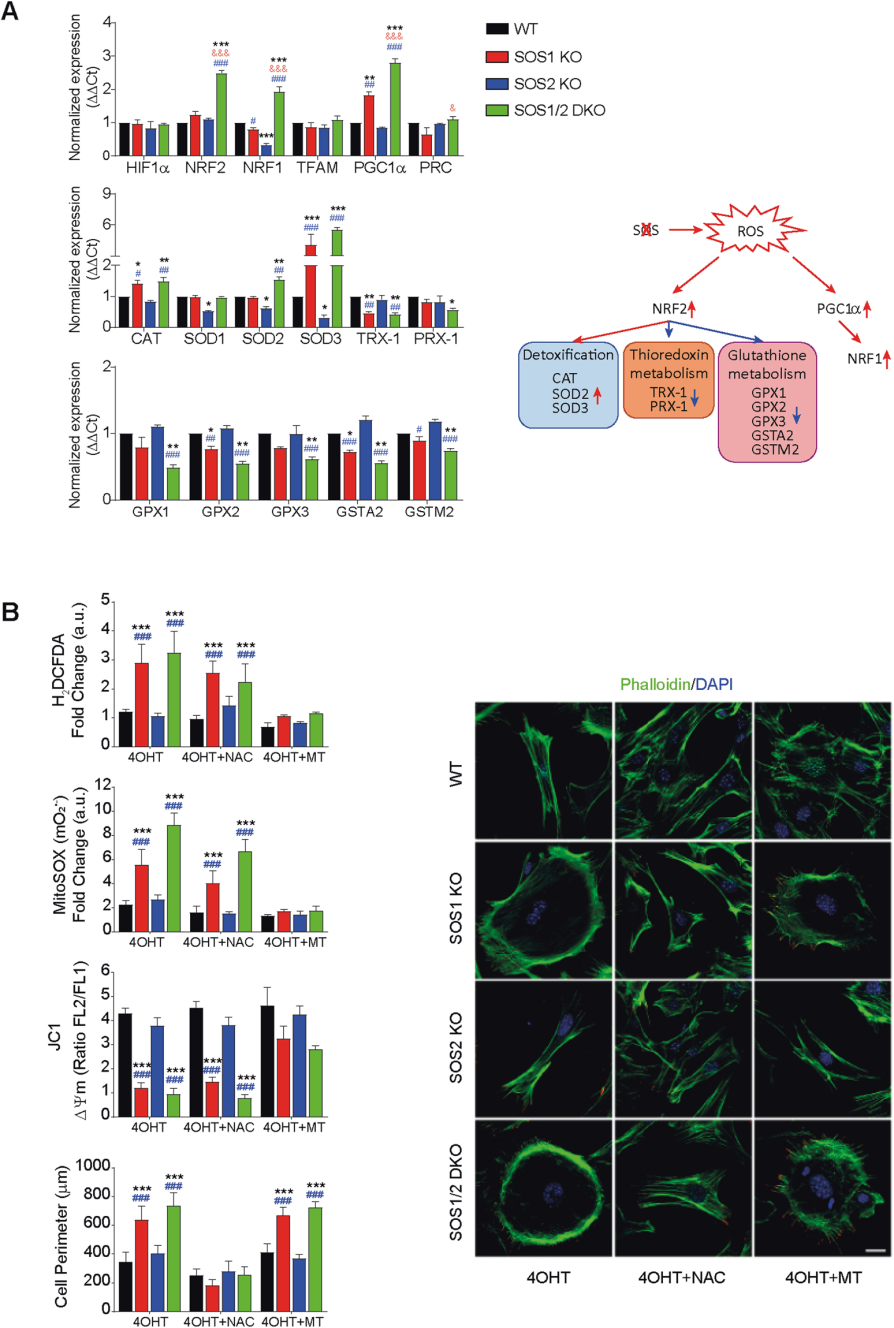
Altered redox phenotypes of SOS1-devoid MEFs (A) and their reversion by antioxidants (B). **A** Expression of cellular antioxidant response elements in MEFs of the four relevant SOS1/2 genotypes. mRNA expression levels of the indicated transcription factors known to participate in hypoxia or antioxidant homeostatic responses (HIF1α, NRF2), mtDNA replication (NRF1, TFAM), or mitochondrial biogenesis (PGC1α), as well as antioxidant and detoxifying enzyme isoforms (CAT, SOD, TRX, PRX, GPX, GST) were determined by quantitative RT-PCR analysis of RNA samples extracted from MEFs of the four relevant SOS genotypes (WT, SOS1-KO, SOS2-KO, SOS1/2-DKO). The expression levels of β-2-microglobulin and β-actin were used as internal controls for normalization in all cases. Sequence of the oligonucleotide primers used in RT-qPCR assays are shown in Supplementary Table S1. A schematic representation of antioxidant pathways depicting the participation in those pathways and the alterations of expression undergone by those players in the absence of SOS1 is also presented. Data represent the mean ± SEM resulting from five independent experiments. * vs WT; ^&^ vs SOS1-KO; ^#^ vs SOS2-KO; ***^,^^&&&^^,^^###^*p* < 0.001; **^,^^##^*p* < 0.01; *^,^^&^^,^^#^*p* < 0.05 (*n* = 5). **B** Rescue of altered redox phenotypes by NAC and MitoTEMPO superoxide scavengers. Primary MEFs of the four relevant SOS genotypes (WT, SOS1-KO, SOS2-KO, SOS1/2-DKO, color-coded as indicated) were left untreated (similar 4OHT tamoxifen treatment for SOS1-KO induction applied to all genotypes to discard off-target effects) or treated with 10 mM NAC (4OHT + NAC) or 100 µM MitoTEMPO (4OHT + MT) as described in Materials and Methods. Left: in vivo quantitation of redox parameters carried out by means of FACS fluorescence measurements performed (10,000 events in each case) on 9-day 4OHT-treated MEFs cultures using specific fluorophores for intracellular ROS (H_2_DCFDA, 5 μM), mitochondrial superoxide^-^(MitoSOX^TM^, 5 μM), and mitochondrial membrane potential (ΔΨm) (JC1, 3 μM) as described in Materials and Methods. Y-axis units in the bar plots represent normalized values calculated as the ratio between the MFI signals measured in MEFs cultured in the presence of 4OHT (SOS1-depleted) and the same MEFs cultured in the absence of 4OHT (SOS1 being expressed). Data are expressed as mean ± SEM of four different experiments (*n* = 4). Right: representative confocal microscopy images of MEFs of the four relevant genotypes co-stained for Phalloidin (green) and DAPI (blue). Scale bar: 25 μm. For quantitation of the changes of cell perimeter caused by antioxidants, we measured individual cells of the four relevant genotypes after growing on culture dishes for 9 days in 4OHT-supplemented DMEM medium in the absence (4OHT) or the presence of NAC (4OHT + NAC) or MitoTEMPO (4OHT + MT). 300 individual cells per genotype were measured in each of six separate experiments (*n* = 6). Statistics: * vs WT; ^#^ vs SOS2-KO. ***^,^^###^*p* < 0.001.

To gain mechanistic insights on these SOS1-dependent phenotypes we first analyzed the expression of known components of processes of ROS detoxification in MEFs of four relevant SOS genotypes (WT, SOS1-KO, SOS2-KO, SOS1/2-DKO) (Fig. 1A and Supplementary Table S1). Interestingly, PPARγ coactivator-1-alpha (PGC1α), a master regulator of mitochondrial respiration and ROS-detoxifying processes [21, 22], was significantly overexpressed in both SOS1-KO and SOS1/2 DKO MEFs. Consistently, other downstream elements of the antioxidant response including nuclear respiratory factors 1 and 2 [23] were also significantly increased in DKO MEFs, whereas no significant changes of other response elements including hypoxia-inducible factor 1-alpha [24], peroxisome proliferator-activated receptor gamma coactivator-related protein 1 [25], or transcription factor A, mitochondrial [26] were detected (Fig. 1A, up). We also detected overexpression, in SOS1-KO and/or SOS1/2 DKO MEFs, of transcripts coding for intracellular catalase (CAT) [27], mitochondrial superoxide dismutase 2 (SOD2), and the extracellular SOD3 superoxide dismutase isoform [28]. On the other hand, no changes of cytoplasmic SOD1 [29] were detected and small but reproducible reduction of transcripts for Thioredoxin H1 and mitochondrial Peroxiredoxin [30] was measured in SOS1-deficient MEFs (Fig. 1A, middle). Finally, we also observed in SOS1-deficient cells a consistently reduced expression of other antioxidant response elements including the GPX, GPX2, and GPX3 isoforms of Glutathione peroxidase [31] or the GSTA2 and GSTMu2 forms of Glutathione S-transferase [32] (Fig. 1A, lower row)

We also carried out detailed comparisons between the effects produced by a general cellular antioxidant like N-acetyl cysteine (NAC) [33] and a mitochondria-targeted antioxidant like MitoTEMPO [29, 34] on the alterations of cellular shape and redox status that are specifically linked to SOS1 depletion (Fig. 1B). Interestingly, these comparisons clearly demonstrated that NAC was able to reverse the morphological changes displayed by SOS1-lacking cells (Fig. 1B, right) but could not reverse the altered redox parameters (Fig. 1B, left) of SOS1-deficient cells. In contrast, treatment with MitoTEMPO did not reverse the morphological defects of SOS1-deficient cells (Fig. 1B, right), but clearly reverted all the oxidative stress phenotypes including elevated levels of intracellular H_2_O_2_ (H_2_DCFDA probe) or mitochondrial superoxide (MitoSOX^TM^ probe) and reduced levels of mitochondrial membrane potential (JC1 probe) of SOS1-KO and SOS1/2-DKO MEFs (Fig. 1B, left panel). It is worth mentioning that the similar 4-Hydroxytamoxifen (4OHT) treatment applied to all MEF genotypes ensured that any redox differences observed among them were specifically due to SOS1 absence. Consistently, we also showed previously [17] that increased oxidative stress correlates with SOS1 loss using different systems for SOS1 ablation (adenoCRE viruses, shRNA) in the absence of 4OHT.

### SOS1 ablation causes specific alterations of mitochondrial morphology, mass, and dynamics

**Fig. 2:**
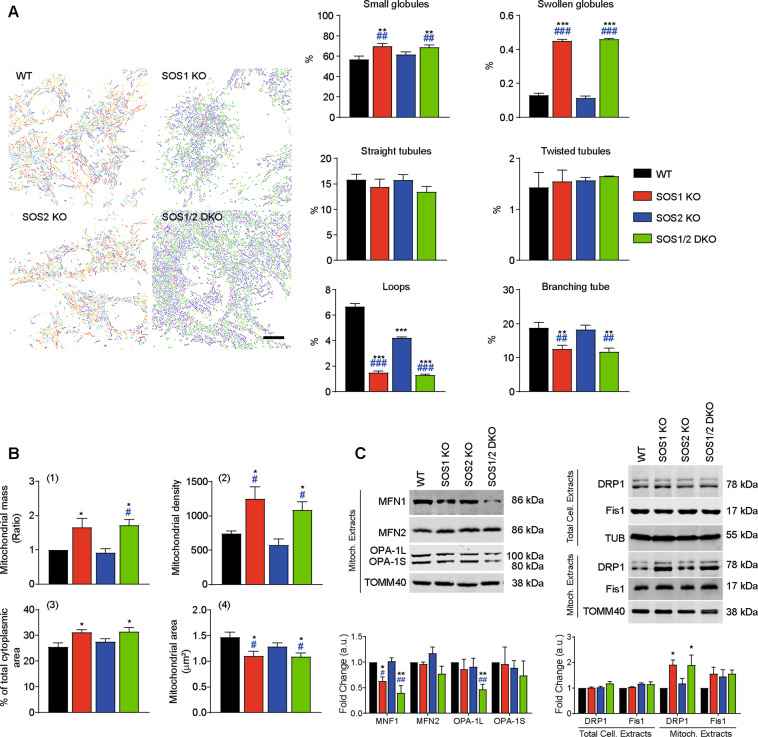
Specific alterations of mitochondrial shape, mass, and dynamics in SOS1-deficient MEFs. **A** Quantitation of mitochondrial morphology subtypes in MEFs of the four relevant SOS genotypes. Left: representative microscopy images of MEFs of the defined genotypes that were immunostained for TOMM40. Immunofluorescence images were filtered and thresholded to obtain segmented images using MicroP tool as described in Materials and Methods. Individual mitochondria were classified according to their shape and size and colored by the software as follows: blue: small globules; yellow: large globules; green: simple tubules; orange: twisted tubules; red: loops; purple: branching tubules. Scale bar: 25 μm. Right: percentage of mitochondrial subtypes of individual cells given different genotypes. In all, 400 individual cells per genotype were measured in each of nine separate experiments. Data expressed as mean ± SEM. Statistical * vs WT; ^#^ vs SOS2-KO. ***^,^^###^*p* < 0.001 **^,^^##^*p* < 0.01 (*n* = 9). **B** Alterations of mitochondrial mass and size in MEFs of the four relevant SOS genotypes. Flow cytometry analysis performed using MitoTracker^TM^ Green (for estimation of Mitochondrial Mass) and TOMM40 antibody (for imaging analysis of mitochondrial structures per cell, distribution and size). **1** Mitochondrial mass: the fluorescence intensity of 10,000 cell stained in vivo with MitoTracker^TM^ Green was quantified by flow cytometry. The total mass of mitochondria was estimated based on MIF values normalized to WT. Data are the mean ± SEM. **2**–**4** Cells fixed and immunostained with TOMM40 antibody were analyzed with Image J software to quantitate different parameters as shown. Mitochondrial density: number of mitochondrial structures per cell; percentage of total cytoplasmic area occupied by mitochondria in the cells. Mitochondrial area: mean area occupied by each individual mitochondrial structure in the cells. Data represent the mean ± SEM of ten sets of experiments. Statistics: * vs WT; ^#^ vs SOS2-KO. *^,^^#^*p* < 0.05 (*n* = 10). **C** Altered levels of mitochondrial fusion and fission regulators in MEFs of the four relevant SOS genotypes. Representative western immunoblots using specific antibodies against mitochondrial MFN1, MFN2, OPA1 (fusion markers) as well as DRP1 and FIS1 (fission markers) in total cellular extracts (total cell extracts) or mitochondrial extracts (mitoch. extracts) from MEFs of the indicated genotypes. TOMM40 and Tubulin were used as loading controls, respectively. Data expressed relative expression vs WT as mean ± SEM. Statistics: * vs WT; ^#^ vs SOS2-KO; **^,^^##^*p* < 0.01; *^,^^#^*p* < 0.05 (*n* = 8).

**Fig. 3:**
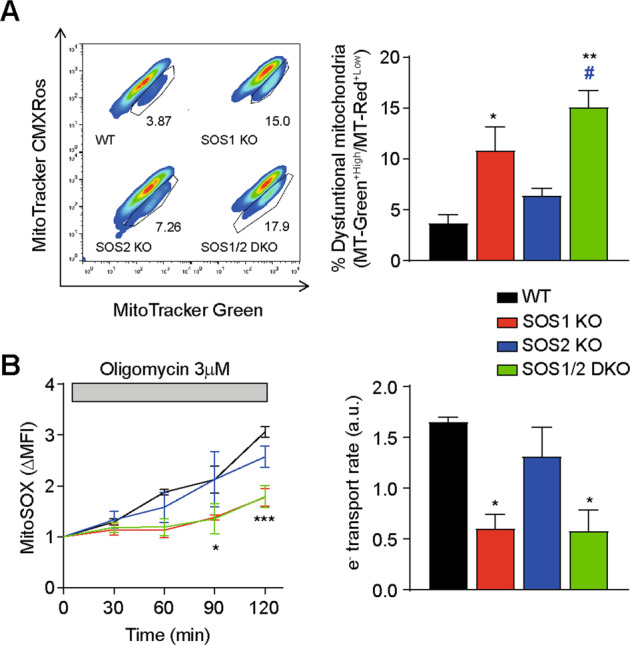
Increased population of dysfunctional mitochondria in SOS1-deficient MEFs. **A** FACS evaluation of mitochondrial membrane functionality. MEFs of the indicated SOS genotypes that were stained with mitochondrial probes MitoTracker^TM^ Green (Δψm-independent) and MitoTracker^TM^ Red CMXRos (Δψm-dependent). Left: representative dotplots and gating strategy used for quantitation of functional (MitoTracker Green^*high*^ and MitoTracker Red^*high*^) and dysfunctional (MitoTracker Green^*high*^ and MitoTracker Red^*low*^) mitochondria in primary MEFs. CCCP (10 μM) was included as a positive control of dysfunctional mitochondria in all experiments. Right: quantitation of percentages of functional and dysfunctional mitochondria in MEFs resulting. Data represented as the mean ± SEM from four separate experiments. Statistics: * vs WT; ^#^ vs SOS2-KO. **p < 0.01; *^,^^#^*p* < 0.05 (*n* = 4). **B** Kinetics of mitochondrial superoxide production and ETC electron transport rate. Primary MEFs of the indicated genotypes were loaded with MitoSOX^TM^ and analyzed by flow cytometry before and after treatment with 3 μM oligomycin to inhibit the ATP synthase, thus forcing electrons produced by the ETC to form superoxide at a rate proportional to the rate of electron transport [44]. Left: normalized MitoSOX^TM^ signal (ΔMFI) over time, basal MitoSOX^TM^ signal (ΔMFI) before oligomycin. Right: electron transport rate as the rate of superoxide production after oligomycin, calculated from the slope of the kinetics curves on the left panel. Data represented as the mean ± SEM from four separate experiments. Statistics: * vs WT. ***p < 0.001; *p < 0.05 (*n* = 4).

**Fig. 4:**
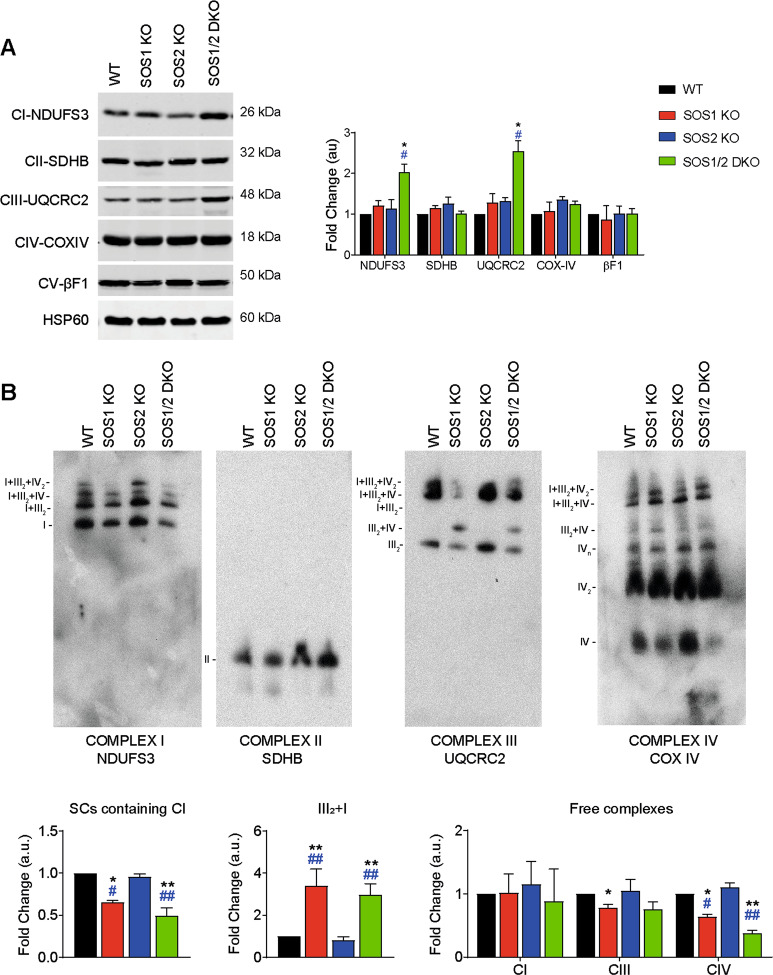
Specific alterations of components of respiratory complexes and assembly into mitochondrial supercomplexes in SOS1-deficient MEFs. Cellular and mitochondrial extracts from 4OHT-treated MEFs (9 days) of the four genotypes were analyzed by WB using antibodies recognizing the indicated components of the mitochondrial complexes. **A** WB quantitation of various protein marker components of mitochondrial supercomplexes in MEFs of the four relevant SOS genotypes. Representative western blot assays of total cell extracts showing the expression of mitochondrial complexes I (NDUFS3), II (SDHB), III (UQCRC2), IV (COX IV), and V (βF1-ATPase). HSP60 was used as specific mitochondrial loading control. Expression levels quantitated as fold change relative to expression in WT samples. Data presented as mean ± SEM. Statistics: * vs WT; ^#^ vs SOS2-KO; *^,^^#^*p* < 0.05 (*n* = 8). **B** Blue Native gel characterization of mitochondrial supercomplexes in MEFs of the relevant SOS genotypes. Representative BN-immunoblots of mitochondrial membrane proteins immunoblotted with antibodies against the indicated subunits of the OXPHOS complexes. The migration of complexes I, I + III_2_, I + III_2_ + IV, and I + III_2_ + IV2 (NDUFS3); complex II (SDHB); complex III_2_, III_2_ + IV, I + III_2_, I + III_2_ + IV, and I + III_2_ + IV_2_ (UQCRC2), and complex IV, IV_2_, IV_n_, III_2_ + IV, I + III_2_ + IV, and I + III_2_ + IV_2_ (COX IV) is indicated on the blots side. All data were normalized by amount of SDHB and represented as fold change in relation to WT. Data presented as mean ± SEM. Statistics: * vs WT; ^#^ vs SOS2-KO; *^,^^#^*p* < 0.05. **^,&&^^,^^##^*p* < 0.01 (*n* = 4).

Using microscopy images of cells immunostained with antibodies against the specific mitochondrial protein TOMM40 [35], we quantified different morphological mitochondrial subtypes in MEFs of the four relevant SOS genotypes [36]. Interestingly, these measurements uncovered specific increase of globular mitochondrial subtypes and decrease of tubular subtypes in SOS1-deficient MEFs (SOS1-KO and SOS1/2-DKO) as compared to WT and SOS2-KO MEFs (Fig. 2A). Quantitation of the mitochondrial immunofluorescence signals [35] also revealed a clear increase of the number of individual mitochondrial structures and the total cytoplasmic area occupied by those structures in the MEFs cytoplasm; consistently, the average size of those individual mitochondrial structures was reduced in SOS1-deficient MEFs as compared to WT and SOS2-KO MEFs (Fig. 2B-2–4). Quantitative FACS analysis of MEFs of the four genotypes loaded with MitoTracker^TM^ Green (fluorophore binding to all mitochondrial structures independently of membrane potential) also revealed significant increase of the overall mitochondrial mass in SOS1-deficient MEFs (SOS1-KO and SOS1/2-DKO) as compared to WT or SOS-KO counterparts grown under the same culture conditions (Fig. 2B-1).

The average mitochondrial morphology results from a dynamic balance between fusion and fission [37]. Immunoblot analyses using antibodies against mitochondrial fusion and fission regulators [37–39] detected in SOS1-deficient MEFs a specific reduction of fusion-promoting proteins such as Mitofusin 1 (MFN1) [40] and long form of mitochondrial dynamin-like GTPase (OPA-1L) [41] as well as unchanged levels of fission promoters such as GTPase dynamin-related protein 1 (DRP1) and Mitochondrial fission 1 protein (FIS1) [42] in total cell extracts. Furthermore, we also detected increased DRP1 expression in purified mitochondrial preparations (Fig. 2C). These observations suggest displacement of the balance toward mitochondrial fission in SOS1-deficient MEFs.

### SOS1 ablation causes specific accumulation of dysfunctional mitochondria

To compare the functionality of the mitochondrial populations of MEFs of the four relevant SOS genotypes, we first evaluated the relative levels of dysfunctional mitochondria populating the cells of the different SOS genotypes (Fig. 3). We used FACS quantitation of the ratios between the fluorescence signals caused after incubation with MitoTracker^TM^ Green (Δψm-independent mitochondrial stain) or MitoTracker^TM^ Red CMXRos (Δψm-dependent mitochondrial stain), so as to distinguish between respiring mitochondria and non-respiring (dysfunctional) mitochondria [43]. Consistent with an increased population of dysfunctional mitochondria associated with SOS1 ablation in MEFs, these data showed significant (≥2-fold), specific increase of the ratio MitoTracker Green^+*high*^/MitoTracker Red^+*low*^ in SOS1-KO and SOS1/2-DKO cells as compared to WT or SOS2-KO MEFs (Fig. 3A).

The dysfunction in the mitochondrial population of SOS1-deficient cells was clearly visible using an experimental approach [44] allowing to compare rates of mitochondrial electron transport in MEFs of the four relevant genotypes (Fig. 3B). In this setting, the electron transport rates were estimated from FACS recordings of the fluorescence signals emitted by MEFs loaded with the superoxide-sensitive, mitochondria-targeted fluorophore MitoSOX^TM^, which allowed monitoring the kinetics of superoxide production after inhibition of the ATP synthase with oligomycin [44]. These data showed markedly reduced rate of superoxide production in SOS1-KO and SOS1/2-DKO mitochondria, confirming a significant loss of mitochondrial functionality linked to SOS1 disappearance in MEFs (Fig. 3B).

### SOS1 ablation is linked to specific alterations of components of mitochondrial respiratory complexes and their assembly in supercomplexes

We also evaluated the expression of representative subunits of the OXPHOS respiratory complexes located in the inner mitochondrial membrane [45] of MEFs of the relevant SOS genotypes. Comparisons of WB immunoblot profiles of mitochondrial extracts detected the specific increase in mitochondria of SOS1-KO MEFs, and especially SOS1/2-DKO MEFs, of the NDUSF3 (NADH dehydrogenase [ubiquinone] iron-sulfur protein 3) subunit of complex I [46] and the UQCRC2 (Cytochrome b-c1 complex subunit 2) subunit of complex III [47], whereas no changes were detected in the level of the SDBH (Succinate dehydrogenase [ubiquinone] iron-sulfur subunit) of complex II [48], the COX IV (Cytochrome c oxidase subunit 4 isoform 1) subunit of complex IV [49], and the F1-beta-subunit of the mitochondrial ATP synthase of complex V [50] (Fig. 4A). As respiratory complexes I and III are the main superoxide producers within mitochondria [51], these observations are mechanistically consistent with the increased levels of superoxide and respiratory stress occurring specifically in SOS1-deficient MEFs.

Formation and assembly of higher order mitochondrial supercomplexes in our SOS-deficient MEFs was evaluated using Blue native gels [52] (Fig. 4B). Since ATP synthase (complex V) and SDHB (complex II) do not participate in mitochondrial supercomplex formation in mammals [53] and showed similar expression levels in all different SOS genotypes analyzed (Fig. 4A), the WB signals of SDHB (complex II) provide adequate internal loading controls to compare the results corresponding to each SOS genotype in the different panels of Fig. 4B. Interestingly, these analyses showed that the SOS1-KO and SOS1/2-DKO samples exhibited a specific, significantly reduced formation of supercomplexes containing complex I (CI and supercomplexes I + III_2_; I + III_2_ + IV; I + III_2_ + IV_2_) in comparison to the WT and SOS2-KO genotypes. The SOS1-deficient samples also showed specific formation of a complex III_2_ + IV released from CI that is absent in WT and SOS2-KO samples, as well as specifically reduced levels of free complex IV (Fig. 4B). These data suggest a specific requirement of SOS1 for correct assembly of mitochondrial supercomplexes.

### Mitochondrial respiratory and metabolic defects specifically linked to SOS1 ablation

**Fig. 5:**
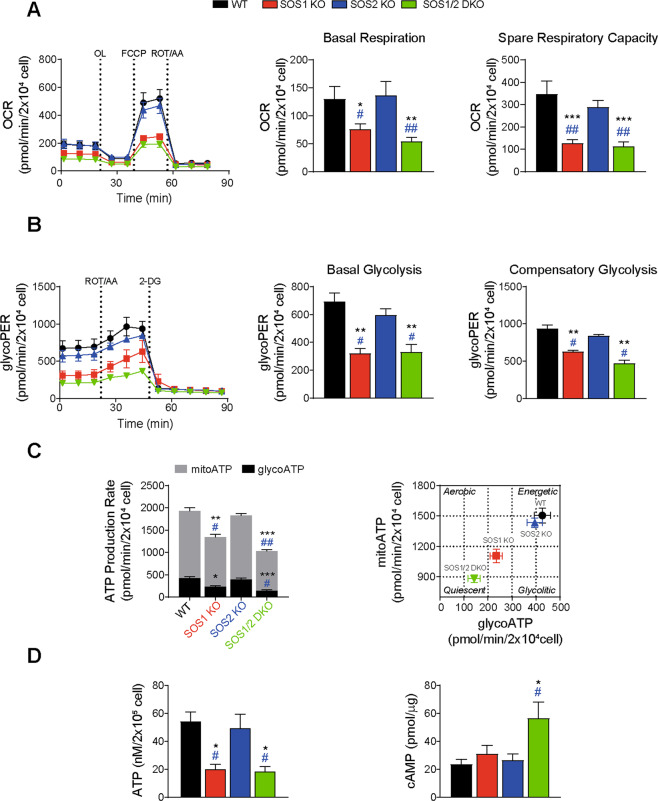
Specific respiratory and metabolic profiles of SOS1-deficient MEFs. **A** Respiration rates. Left: Seahorse XF Cell Mito Stress Test performed on primary MEFs of the indicated genotypes. 20,000 cells/well were seeded and incubated for 24 h. OCR (oxygen consumption rate) was measured under basal conditions followed by the sequential addition of 1.5 µM Oligomycin (OL), 1 µM FCCP, and 1 µM Rotenone and Antimycin A (ROT/AA) following manufacturer’s instruction. Right: quantitation of parameters for basal respiration and spare respiratory capacity. Data presented are the mean ± SEM from six independent experiments using at least five technical replicates per experiment per condition. Statistics: * vs WT; ^&^ vs SOS1-KO; ^#^ vs SOS2-KO; ***^,^^###^*p* < 0.001; **^,&&^^,^^##^*p* < 0.01; *^,^^&^*p* < 0.05 (*n* = 6). **B** Glycolytic rates. Left: Seahorse XF glycolytic rate assay performed on primary MEFs of the indicated genotypes. 20,000 cells/well were seeded and incubated for 24 h. ECAR (extracellular acidification rate) was measured under basal conditions followed by the sequential addition of 1 µM Rotenone and Antimycin A (ROT/AA), and 100 mM 2-deoxyglucose (2-DG) following the manufacturer’s instructions. Right: quantitation of glycolytic proton efflux rate (glycoPER) and individual parameters for basal glycolysis and compensatory glycolysis. Data expressed as the mean ± SEM compiled from five independent experiments using at least five technical replicates per experiment per condition. Statistics: * vs WT; ^#^ vs SOS2-KO; **p < 0.01; ^#^*p* < 0.05 (*n* = 5). **C** ATP production rates. Left: Seahorse XF real-time ATP rate assays of primary MEFs of the indicated genotypes. Rates of mitochondrial ATP production (mitoATP, gray bars) and glycolytic ATP production (glycoATP, black bars) quantitated following manufacturer’s instruction. Right: energetic map of the four genotypes tested charting mitochondrial ATP (mitoATP) versus glycolysis-generated ATP (glycoATP). Data shown are compiled from five independent experiments using at least five technical replicates per experiment per condition. Statistics: * vs WT; ^#^ vs SOS2-KO; ***p < 0.001; **^,^^##^*p* < 0.01; *^,^^#^*p* < 0.05 (*n* = 5). **D** Intracellular ATP and cAMP levels. Left: measurements of total ATP content (normalized by cell number) in cellular lysates. Right: intracellular cAMP levels measurements (normalized by μg protein) in total cellular lysates. Data shown as the mean ± SEM compiled from eight independent experiments using at least three technical replicates per experiment per genotype. Statistics: * vs WT; ^#^ vs SOS2-KO; *^,^^#^*p* < 0.05 (*n* = 8).

To ascertain whether the specific mitochondrial defects of SOS1-deficient cells were also reflected in related functional alterations, we analyzed the respiratory and metabolic profiles [54] of SOS1-deficient MEFs (Fig. 5). OCR measurements detected significantly reduced rate of basal respiration, and particulary spare respiratory capacity, in SOS1-deficient MEFs. Consistent with previous observations [17] SOS1/2-DKO MEFs showed significantly worse respiratory parameters than single SOS1-KO MEFs in these assays (Fig. 5A). Glycolysis constitutes an additional source of cellular energy in addition to oxidative phosphorylation [55] and our measurements of basal glycolytic rates and compensatory glycolytic rates also identified significant inhibition of both parameters in SOS1-deficient MEFs (Fig. 5B). Consistently, we also detected significantly decreased rates of ATP production from glycolysis (glycoATP) and oxidative phosphorylation (mitoATP) in specific association with SOS1 disappearance in MEFs (Fig. 5C). A plot correlating the MEF genotypes with their respective rates of mitoATP and glycoATP production clearly discriminated the profiles of WT and SOS2-KO MEFs from that of SOS1-KO and SOS1/2-DKO MEFs, which presented significantly less energetic and more quiescent phenotypic profiles (Fig. 5C right). Direct luminescence measurements of cultured MEFs confirmed the reduction of ATP production in SOS1-deficient MEFs and also revealed a significant increase of intracellular cAMP concentration in SOS1/2 DKO MEFs (Fig. 5D).

### Specific alterations of substrate oxidation capabilities in SOS1-deficient MEFs

**Fig. 6:**
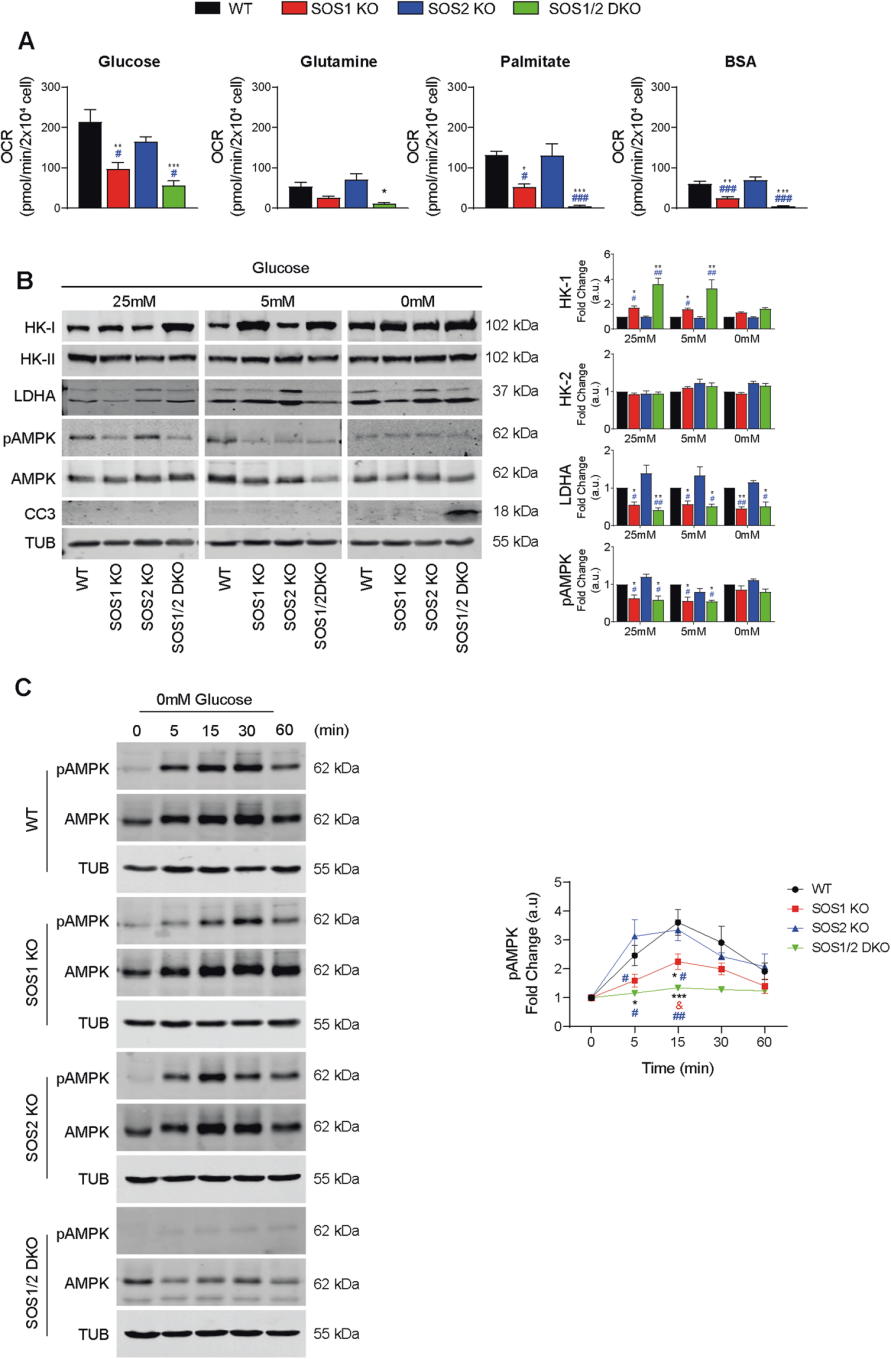
Specific patterns of utilization of oxidation substrates in WT and SOS-deficient MEFs. **A** Rate of oxidation of different substrate nutrients under conditions of high substrate demand. Seahorse XF Cell Mito Stress Test assays were performed on primary MEFs of the indicated genotypes that had been starved overnight and then treated as described in Materials and Methods under specific conditions designed for testing their capability of oxidation of glucose, glutamine, palmitate, or endogenous lipids (BSA) as the only available respiratory substrate. OCR was measured under basal conditions or under previous 1 h treatment with UK5099 (20 µM, 1 h) for glucose utilization test, the GSL1 inhibitor BPTES (20 µM, 1 h) for glutamine utilization tests, or Etomoxir (100 µM 1 h) for tests of utilization of exogenous fatty acids (Palmitate) or (BSA), followed by the sequential addition of 1.5 µM Oligomycin (OL), 1 µM FCCP, and 1 µM Rotenone and Antimycin A (ROT/AA). Bars in the graphs represent the result of subtracting the absolute values of maximal respiration rates (MRC) obtained in the presence of the indicated inhibitors from the absolute MRC values obtained in the absence of the inhibitors. The actual OCR tracings and graph bars corresponding to the different assays of glucose, glutamine, or fatty acid utilization are shown in Supplementary Fig. S1. Results compiled from six independent experiments using at least five technical replicates per experiment per genotype. Values expressed as mean ± SEM. * vs WT; ^#^ vs SOS2-OK; ***^,^^###^*p* < 0.001; **p < 0.01; *^,^^#^*p* < 0.05 (*n* = 6). **B** Altered expression patterns of glycolytic enzymes in SOS1-KO and SOS1/2-DKO cells. Cells were cultured in DMEM medium with different concentrations of glucose for 24 h and cellular extracts were then analyzed by WB. Representative western blots (left) and densitometric analyses (right) of the expression of Hexokinase-I (HK-I), Hexokinase II (HK-II), LDHA, pAMPK, and cleaved caspase-3 (CC3) in cells of the indicated genotypes cultured in the presence of the indicated glucose concentrations. Tubulin (TUB) used as internal loading control for normalization of expression levels in all cases. Data resulting from five independent experiments performed. Values expressed as mean ± SEM. * vs WT; ^#^ vs SOS2-KO; **^,^^##^*p* < 0.01; *^,^^#^*p* < 0.05 (*n* = 5). **C** Altered kinetics of pAMPK signaling upon glucose deprivation in SOS1-deficient cells. Representative western blot (left) and densitometric analyses (right) of phosphorylated AMPK expression upon glucose deprivation in MEFs cultures of the indicated genotypes. AMPK was used for normalization of fold change values (a.u.) and Tubulin (TUB) was used as internal loading control. Data resulting from five independent experiments performed. Values were expressed as mean ± SEM. * vs WT; ^#^ vs SOS2-KO; ***p < 0.001; **^,^^##^*p* < 0.01; *^,^^#^*p* < 0.05 (*n* = 8).

As different cell types utilize a variety of nutrient substrates to support oxidative energy metabolism via TCA and the ETC in mitochondria [56], we wished to ascertain whether the absence of SOS1 could also impact the type of preferred substrates or their mechanisms of oxidation in MEFs. We first compared OCR profiles of MEFs of the four relevant SOS genotypes cultured under conditions where only one of three alternative oxidation substrates— namely, glutamine, fatty acids (palmitate), or glucose—was available [44]. These assays detected distinctive, specific patterns of impairment of substrate oxidation capabilities in SOS1-deficient MEFs as compared to WT and SOS2-KO MEFs (Fig. 6A). Regarding glutamine utilization, the OCR tracings revealed very low oxidation rates for all genotypes tested, and the rates measured in SOS1-deficient MEFs were even more reduced or almost negligible as compared to WT and SOS2-KO cells (Fig. 6A). A similar pattern of almost negligible oxidation rates occurred in SOS1-deficient MEFs under conditions where only endogenous fatty acids (with exogenously added BSA) were available for oxidation. On the other hand, sizeable OCR rates of oxidation of exogenously added fatty acids (palmitate) could be measured in all MEF genotypes although the SOS1-KO MEFs showed more than 50% reduction and the SOS1/2-DKO MEFs showed almost negligible OCR values in comparison to the other two genotypes (Fig. 6A). Finally, the OCR tracings for glucose utilization clearly indicated that this is the preferred oxidation substrate for MEFs and also showed that SOS1-deficient MEFs can indeed use glucose as the only respiratory substrate (Fig. 5B), but they do it much less efficiently (~50% reduction) than their WT or SOS2-KO counterparts (Fig. 6A and Supplementary Fig. S1).

We also compared the expression patterns of various glucose-metabolizing enzymes in MEFs cultured in the absence or presence of limiting glucose concentrations for 24 h. In the absence of glucose (0 mM), we were able to detect cleaved caspase-3 (CC3) only in SOS1/2-DKO MEFs suggesting a need of the GEF activity provided by either SOS isoform to prevent cell death and support MEF survival after glucose deprivation (Fig. 6B). In the presence of glucose (5 and 25 mM), the SOS1-KO and SOS1/2-DKO MEFs displayed a specific increase of hexokinase-I HK1 (but not HK2) [57] and specific decrease of phospho-AMP-activated protein kinase (pAMPK) [58] and lactate dehydrogenase A (LDHA) (also in 0 mM) in comparison to the other genotypes (Fig. 6B). We also observed a significantly impaired response of SOS1-deficient MEFs regarding the kinetics of pAMPK expression [58] in response to elimination of glucose from the growth medium (Fig. 6C).

### Defective respiratory and metabolic phenotypes of RASless cells

**Fig. 7:**
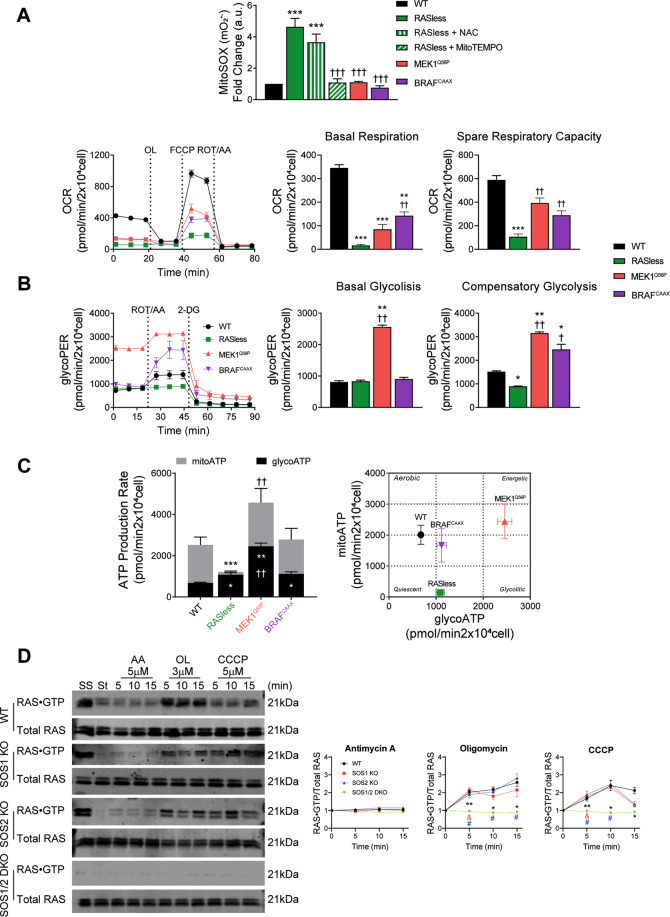
Specific respiratory and metabolic phenotypes of RASless cells. **A** Comparison of redox parameters of WT and RASless MEFs under different experimental conditions. MEF cultures of the indicated genotypes and color codes (control WT cells, black; RASless (H-RAS^−/−^;N-RAS^−/−^;K-RAS^lox/lox^) cells, green; RASless cells expressing transfected MEK1^Q56P^, red; and RASless cells expressing transfected BRAF^CAAX^, purple) were grown for 12 days in the absence or presence of 4OHT as indicated in each case and tested for various redox, metabolic, and mitochondrial phenotypes as shown here. Upper row: quantitation of mitochondrial superoxide O_2_•– production. FACS fluorescence measurements performed (10,000 events in each case) using the specific mitochondrial fluorophore MitoSOX^TM^ (5 μM) as described in Materials and Methods. The effect of treatment of RASless cells with a general antioxidant (10 mM NAC, green vertical lines) and a specific mitochondrial superoxide scavenger (100 µM MitoTEMPO, green tilted lines) was also tested. Units in the Y-axis represent normalized values calculated as the ratio between the MFI signals produced by each specific MEF cell line after growing in the presence of 4OHT (KRAS-depleted) or in the absence of 4OHT (KRAS still being expressed). Lower row: measurement of respiratory rates. Left: Seahorse XF Cell Mito Stress Tests performed on MEF cultures of the indicated genotypes after growing for 12 days in the presence of 4OHT. 20,000 cells/well were seeded and incubated in Seahorse testing cartridges for 24 h before performing OCR measurements under basal conditions followed by the sequential addition of 1.5 µM Oligomycin (OL), 1 µM FCCP, and 1 µM Rotenone and Antimycin A (ROT/AA). Right: quantitation of parameters for basal respiration and spare respiratory capacity. Data presented are the mean ± SEM from five independent experiments using at least five technical replicates per experiment per condition. Statistics: * vs WT; ^†^ vs RASless; ***^,†††^*p* < 0.001; **^,††^*p* < 0.01.(*n* = 5). **B** Glycolytic rates. Left: Seahorse XF glycolytic rate assays performed on WT and RASless MEFs. 20,000 cells/well were seeded and incubated for 24 h in Seahorse microplates before measuring ECAR under basal conditions followed by the sequential addition of 1 µM Rotenone and Antimycin A (ROT/AA), and 100 mM 2-deoxyglucose (2-DG). Right: quantitation of glycolytic proton efflux rate (glycoPER) and individual parameters for basal glycolysis and compensatory glycolysis of cells. Data expressed as mean ± SEM and compiled from six independent experiments using at least five technical replicates per experiment per genotype. Statistics: * vs WT; ^†^ vs RASless; **^,††^*p* < 0.01; *^,†^*p* < 0.05 (*n* = 6). **C** ATP production rates. Left: Seahorse XF real-time ATP production rate tests performed on WT and RASless MEFs. 20,000 cells/well were seeded and incubated for 24 h in Seahorse microplates before measuring ECAR (for estimation of glycoATP under basal, untreated conditions) and OCR (for estimation of mitoATP) followed by the sequential addition of 1 µM Rotenone and Antimycin A (ROT/AA), and 100 mM 2-deoxyglucose (2-DG). Right: bioenergetic phenotypic maps charting mitoATP *vs* glycoATP values for each MEF genotype. Data expressed as mean ± SEM and compiled from six independent experiments using at least five technical replicates per experiment per genotype. Statistics: * vs WT; ^†^ vs RASless; ***p < 0.001; **^,††^*p* < 0.01; *p < 0.05 (*n* = 6). **D** RAS activation assays. Representative western blots (left) and densitometric analysis (right) of assays of RAS•GTP formation assays performed in WT and SOS-deficient MEFs in the presence of inhibitors of the electron transport chain. Primary MEFs of the indicated genotypes grown for 9 days in the presence of 4OHT were starved overnight and then treated for the times indicated with the specific inhibitors of the ETC including Antimycin A (AA, 5 µM), Oligomycin (OL, 3 µM), and CCCP (5 µM). Subsequent analysis of RAS•GTP formation was done by means of pull-down assays using beads loaded with the RBD region of RAF and a specific pan-RAS antibody as described in Materials and Methods. The graphs present normalized data of the kinetics of RAS•GTP formation after exposure to the indicated inhibitors, quantitated in each case as the ratio between the densitometric signals corresponding to RAS•GTP complexes and to total RAS in the western immunoblots. Data expressed are the mean ± SEM from six independent experiments performed using at least four different MEF lines per genotype. SS, steady state growing cultures. St, starved cultures. Statistics: * vs WT; ^&^ vs SOS1-KO; ^#^ vs SOS2-KO; **p < 0.01. *^,&,#^*p* < 0.05 (*n* = 6).

To explore potential mechanistic contribution of RAS proteins (the targets of SOS-GEFs) to the defective phenotypes of SOS1less MEFs, we characterized various redox, respiratory and metabolic parameters in quiescent, non-proliferating RASless MEFs devoid of the canonical members of the RAS subfamily, as well as in derived clones that recovered proliferating ability upon expression of exogenously introduced clones of constitutively activated BRAF^CAAX^ or MEK11^Q56P^ [19, 20] (Fig. 7 and Supplementary Fig. S2).

Quantitation of mitochondrial superoxide using the specific fluorophore MitoSOX^TM^ [59] showed significantly increased levels in RASless cells (H-RAS^−/−^;N-RAS^−/−^;K-RAS^lox/lox^) devoid of RAS proteins after treatment with tamoxifen for complete removal of KRAS [19, 20]. Furthermore, as with SOS1-deficient MEFs, treatment with MitoTEMPO, but not with NAC, restored normal superoxide levels to the RASless MEFs. Remarkably, RASless clones that recovered proliferative ability after transfection of activated, downstream RAS signaling elements such as BRAF^CAAX^ or MEK1^Q56P^ showed normal levels of superoxide (similar to MitoTEMPO-treated RASless cells) (Fig. 7A, up). The OCR assays also revealed that the non-proliferating, quiescent RASless cells showed a dramatic decrease of basal respiration and spare respiratory capacity in comparison to normal WT controls. Furthermore, the clones that recovered proliferative ability upon expression of activated MEK1 or BRAF showed significant, although not complete, recovery (~50%) of respiratory parameters, indicating at least partial recovery of mitochondrial function in these clones (Fig. 7A, lower row).

RASless cells exhibited similar rates of basal and compensatory glycolysis than WT controls. On the other hand, only MEK1-rescued cells, but not BRAF-rescued MEFs, showed significantly elevated rescued level of basal glycolytic proton efflux rates (glycoPER), whereas both MEK1^Q56P^-rescued and BRAF^CAAX^-rescued cells showed significantly increased rates of compensatory glycolysis in comparison to WT or RASless cells (Fig. 7B). These observations suggest that different compensatory mechanisms may mediate the recovery of proliferative ability in RASless cells expressing either MEK1^Q56P^ or BRAF^CAAX^ [19, 20, 60].

The rates of basal glycolytic ATP production were similar or only slightly elevated in quiescent RASless cells as compared to proliferating WT control MEFs, whereas the proliferation-rescued MEK1^Q56P^ clones showed significantly higher levels of basal glycoATP production than the rest of genotypes (Fig. 7C, black bars). RASless cells showed almost nil rates of mitoATP production in comparison to WT control MEFs (Fig. 7C, gray bars). These analyses showed that the rescue of proliferative ability in BRAF^CAAX^- or MEK11^Q56P^-expressing clones was linked to significant increase of the rate of mitoATP production (compared to WT controls), although the MEK1^Q56P^-rescued MEFs always showed significantly higher levels than the BRAF^CAAX^-rescued cells in the assays (Fig. 7C, left). Plotting the ATP production values in cell energy phenotypic profiles clearly discriminated the metabolic potential of control WT MEFs from the quiescent RASless cells or the proliferation-rescued MEK1^Q56P^ and BRAF^CAAX^ clones, showing that the MEK1^Q56P^-rescued cells exhibit the highest metabolic and energetic potential. This plot also visualized that, as for SOS1less cells, glycolysis is the primary bioenergetic pathway for RASless cells (Fig. 7C, right).

The above observations suggest that defective activation of target RAS proteins (due to absence of the specific RAS-GEF activity of SOS1) might account, at least in part, for the defective redox phenotypes of SOS1-deficient MEFs. In this regard, it is also relevant in our observation that cellular RAS proteins may become activated after treatment of MEFs with various specific inhibitors of the electron transport chain in mitochondria (Fig. 7D). Treatment with antimycin (which causes complete disruption of the ETC and completely blocks ATP production [61]) did not result in any RAS activation in any of the MEF genotypes tested. In contrast, treatment with oligomycin (targeting the ATP synthase but unable to completely inhibit electron flow [62]) or with the CCCP protonophore (which causes uncoupling of the proton gradient, thus reducing the ability of ATP synthase to function optimally [63]) resulted in significant RAS activation (RAS•GTP formation) in single SOS1-KO or SOS2-KO MEFs that was only slightly lower than in WT MEFs. Interestingly, SOS1/2-DKO MEFs did not show any level of RAS activation after treatment with these two inhibitors (Fig. 7D), a behavior paralleling previous observations relative to other SOS1-specific defective phenotypes, which show significantly higher intensity in SOS1/2-DKO cells than in SOS1-KO cells [14, 17, 18].

## Discussion

We reported previously that SOS1 ablation causes defective morphological and functional phenotypes in mouse cells including, in particular, significantly increased levels of intracellular oxidative stress [12–14, 17, 18]. Here we compared redox and metabolic parameters of WT, SOS1-KO, SOS2-KO, and SOS1/2-DKO primary MEFs with an aim at obtaining functional/mechanistic insights regarding the origin of the elevated intracellular oxidative stress detected specifically in SOS1-deficient cells. In this regard, our initial analyses identified a specific expression profile of cellular and mitochondrial molecules constituting the core of components of the antioxidant response [22, 23, 27, 28, 31, 32] triggered specifically in SOS1-ablated cells. It was previously reported that cells harboring KRAS^G12D^ display upregulation of detoxifying enzymes such as PRX, TRX, CAT [64] as well as activation of NRF2 [65] leading to an increase in ROS detoxification capacity. In our SOS-deficient cells we observed increased expression of superoxide detoxifying proteins (SOD2 and SOD3) but we did not observe increase of elements responsible for metabolizing H_2_O_2_. These observations support the notion that RAS activation may be involved in modulation of the antioxidant cellular capacity. More importantly, the clearly differential effects produced by the general antioxidant NAC [33] compared to the mitochondria-targeted MitoTEMPO [29] provided further mechanistic clues regarding the defective cellular phenotypes of SOS1-deficient cells. These observations suggest that different molecular mechanisms underlie the flattened/spread morphology and the elevated ROS shown by SOS1-deficient MEFs and focus attention on a possible prevalent role of mitochondria to generate the oxidative stress suffered by these KO cells.

Characterization of mitochondrial morphological subtypes [36] identified specific increase of globular forms and decrease of tubular forms in the mitochondrial population of Sos1-deficient MEFs. These analyses also showed specific increase of mitochondrial mass [66, 67] and total number of individual mitochondria, together with reduced expression of promoters of mitochondrial fusion [37, 68] in SOS1-deficient MEFs. DRP1 is an essential player in mitochondrial fission and its phosphorylation by specific kinases (including AMPK, MAPK, PKC and CDK1/cyclinB1) is known to induce translocation from the cytosol to the mitochondrial outer membrane [39]. We speculate that increased ROS may activate the ROS/PKC pathway and subsequent phosphorylation of DRP1, thus leading to its increased translocation to mitochondria as observed in Sos1-deficient cells. As mitochondrial morphology and function is the result of a dynamic balance within these organelles, it is apparent that these alterations of mitochondrial morphology, mass, and dynamics represent adaptive responses to the increased oxidative stress [42, 67] that are mechanistically linked to unbalance between fusion and fission in mitochondria of SOS1-deficient MEFs [39, 69, 70]. In contrast to our primary, SOS1-ablated cell lines [12, 14, 17, 18], no significant changes of mitochondrial morphology were detected in the immortalized RASless cell lines [19, 20] analyzed in this report. It will be relevant to ascertain in future whether the immortalization process may mask visibility of potential mitochondrial alterations in immortalized cell lines.

The structural defects of mitochondria in SOS1-deficient MEFs also translated into relevant functional defects. Using fluorophores that are dependent (MitoTracker^TM^ Red CMXROS) or independent (MitoTracker^TM^ Green) on the mitochondrial membrane potential, we detected significant increase numbers of dysfunctional mitochondria and consistent decrease of electron transport rates in mitochondrial preparations [44] of SOS1-ablated cells as compared to WT or SOS2-KO counterparts.

Consistent with the notion that structure and function of mitochondria are inextricably linked [54, 71], SOS1-deficient MEFs showed specific alterations concerning specific subunits of respiratory complexes of the inner mitochondrial membrane and their assembly into mitochondrial supercomplexes [45, 72]. It is likely that the detection of elevated levels of the NDUFS3 subunit of complex I and the UQCRC2 subunit of complex III could be mechanistically linked to the increased superoxide levels and respiratory stress of SOS1-deficient MEFs since complexes I and III are the main superoxide producers in mitochondria [51, 73]. Other consistent alterations detected in SOS1-deficient mitochondria included reduction of supercomplexes containing complex I, which leads to the lack of assembly of complex III_2_ + IV into supercomplex, and reduced levels of free complex IV. Notably, none of these alterations was observed in WT and SOS2-KO cells, which do not present any oxidative stress or mitochondrial/respiratory defects. NDUFS3 is an assembly factor acting in the first steps of the biogenesis of CI and is an essential subunit in CI assembly, stability, and function [74]. The correct assembly of this complex requires the interaction with complexes III and IV [75]. This suggests the possibility that increased expression of NDUFS3 and UQCRC2 might be a compensatory mechanism for SOS1-deficient cells to rescue the assembly of SCs. The defective assembly of respiratory complexes in SOS1-deficient MEFs is also consistent with their higher level of ROS production and lower rates of respiratory activity over different substrates feeding with electrons the respiratory chain [76]. Altogether, these observations indicate that the functional contribution of SOS1 is critical for adequate assembly of mitochondrial supercomplexes in mouse cells.

Oncogenic KRAS is known to regulate metabolism in the tumor environment increasing glucose uptake and glycolysis by directly regulating HK1 [77] activity and increasing LDHA [78] expression. The structural and functional alterations of mitochondria in SOS1-deficient cells were also accompanied by consistent respiratory/metabolic defects. Thus, significantly reduced respiratory and glycolytic rates, together with consistently reduced ATP production rates, were measured in SOS1-deficient MEFs as compared to WT or SOS2-KO counterparts. As a consequence, the metabolic/energetic profiles of WT and SOS2-KO cells can be clearly discriminated from those of the oxidation-stressed SOS1-KO and SOS1/2-DKO cells. SOS1-deficient MEFs also showed specifically reduced capacity to utilize different oxidation substrates in comparison to WT or SOS2-KO controls and significantly higher dependency on glucose for growth and survival than the rest of genotypes, displaying also specifically altered levels of glycolytic enzymes (elevated HK1 [77] and reduced LDHA [78, 79] and pAMPK [58]). Elevated cAMP signaling slows down the import of mitochondrial proteins, and fosters the metabolic switch from OXPHOS to glycolysis [80]. However, the increased cAMP level of SOS1/2 DKO is clearly not enough to restore mitochondrial function and therefore further studies are needed to ascertain the role of the mitochondrial cAMP-PKA pathway in SOS1/2-depleted cells. Together, all our observations suggest a critical contribution of SOS1 function(s) to the molecular mechanisms involved in glucose utilization as oxidation substrate supporting growth and survival of MEFs in culture.

Characterization of redox parameters of RASless cells [19, 20] devoid of the main canonical RAS targets for SOS1-GEF activity [7] provided further mechanistic clues to the defective mitochondrial/respiratory phenotypes of SOS1less MEFs. These analyses uncovered intracellular redox defects that, although not totally coincident (RASless cells displayed even lower mitochondrial respiratory rates and higher glycolytic capacity than SOSless cells), were highly reminiscent of those previously seen in SOS1less MEFs. Anyhow, the similarity of the respiratory/metabolic phenotypes of RASless cells with those of SOS1-KO and SOS1/2-DKO MEFs, and the observation that the intensity of redox defects was always higher in SOS1/2-DKO than in SOS1-KO cells [12, 17, 18] suggests that the mitochondrial phenotypes of SOS1-ablated cells are mechanistically related to defective/missing activation of specific RAS target proteins due to the specific absence of SOS1-GEF activity. This is further supported by the observation that cellular RAS proteins become activated (GTP loaded) after blocking the electron transport chain with mitochondrial inhibitors.

Altogether, our data uncover a direct functional role of SOS1 in control of intracellular and mitochondrial redox homeostasis, and suggest that the activation of RAS proteins by GEF activity of SOS1 is specifically required for correct dynamics and function of mitochondria.

## Materials and methods

### Cell culture

E13.5 MEFs from all genotypes were isolated as previously described [17] and equally treated for 9 days with 4OHT (0.3 μM, Sigma-Aldrich; H6278; in DMEM with 10% FBS and glutamine) to discard any potential off-target effects. Cells were routinely tested for Mycoplasma (PlasmoTest Mycoplasma Detection Kit; InvivoGen, rep-pt1).

### Isolation of mitochondria and Blue Native gel electrophoresis

Cells were homogenized in a glass-Teflon homogenizer with seven volumes of hypotonic buffer (83 mM sucrose, 10 mM MOPS pH 7.2). After homogenization, the same volume of hypertonic buffer (250 mM sucrose, 30 mM MOPS pH 7.2) was added and nuclei and unbroken cells were removed by centrifugation at 1000 *g*. Mitochondria were obtained by centrifugation at 12,000 *g* and washed in buffer A (320 mM sucrose, 1 mM EDTA, 10 mM Tris-HCl pH 7.4). For Blue Native (BN) gels, mitochondrial pellets were resuspended in 50 mM Tris-HCl pH 7.0 containing 1 M 6-aminohexanoic acid at a final concentration of 10 mg/ml. The membranes were solubilized by the addition of 10% digitonin (4:1 digitonin/mitochondrial protein). Then, 5% Serva Blue G dye in 1 M 6- aminohexanoic acid was added to the solubilized membranes. Native PAGE™ Novex^®^ 3–12% Bis-Tris Protein Gels (Life Technologies, BN1001BOX) were loaded with 70 μg of mitochondrial protein. After fractionation, the gels were electroblotted onto PVDF membranes. Membranes were further processed for immunoblotting [52].

### Immunoblotting

All antibodies and conditions used in western blot assays are described in Supplementary Table S2.

### Metabolic flux assays

ECAR and OCR measurement were performed using a Seahorse XFe24 analyzer (Seahorse Bioscience) on MEFs (20,000 cells/well) plated on XFe24 tissue culture plates coated with fibronectin (3 μg/ml). Seahorse XF Cell Mito Stress Test Kit (103015-100), XF Glycolytic Rate Assay Kit (103344-100), XF Real-Time ATP Rate Assay Kit (103592-100), and XF Palmitate Oxidation Stress Test Kit (103693-100) were used according to the manufacturer’s protocol. For OXPHOS experiments testing glucose, glutamine, and fatty acid as respiratory substrate [44], cells were incubated with DMEM minimum medium (without glucose, sodium pyruvate, L-Glutamine, and 1% FBS) 24 h before the assay. One hour prior to measurements, cells were treated/untreated, respectively, with UK5099 (10 µM, Selleckchem, S5317), BPTES (20 µM, Selleckchem, S7753), or Etomoxir (100 µM, Selleckchem, S8244), and incubated (CO_2_-free atmosphere) at 37 °C.

### Statistics

GraphPad Prism 8.0.1 (GraphPad Inc., USA) software was used. All data presented are average of at least four independent experiments performed in triplicates. Results are expressed as mean ± SEM. Differences between experimental groups analyzed using one-way ANOVA and Bonferroni’s tests. No statistical method was used for predetermination of sample size.

## Supplementary information

Supplementary Information
