# Supplementary material for: Critical requirement of SOS1 RAS-GEF function for mitochondrial dynamics, metabolism, and redox homeostasis
Source: Oncogene. 2021 Jun 12;40(27):4538–51. doi: 10.1038/s41388-021-01886-3 (PMC8266680; doi:10.1038/s41388-021-01886-3)
Supplement: Supplementary file 1 — Supplementary Information [file 41388_2021_1886_MOESM1_ESM.pdf]

SUPPLEMENTARY INFORMATION

Critical requirement of SOS1 RAS-GEF function for mitochondrial dynamics, metabolism and redox homeostasis

Rósula García-Navas[1,2], Pilar Liceras-Boillos[1,2], Carmela Gómez[1,2], Fernando C. Baltanás[1,2], Nuria Calzada[1,2] , Cristina Nuevo-Tapioles[3,4], José M. Cuezva[3,4], Eugenio Santos[1,2]

[1]Centro de Investigación del Cáncer-Instituto de Biología Molecular y Celular del Cáncer (CSIC- Universidad de Salamanca), Salamanca, Spain

[2] Centro de Investigación Biomédica en Red de Cáncer - Cáncer (CIBERONC), Madrid, Spain.

[3] Departamento de Biología Molecular, Centro de Biología Molecular Severo Ochoa3, (CSIC-Universidad Autónoma de Madrid), Madrid, Spain

[4] Centro de Investigación Biomédica en Red de Cáncer – Enfermedades Raras (CIBERER), Madrid, Spain, Madrid, Spain.

SUPPLEMENTARY MATERIALS AND METHODS

RT-qPCR. Total RNA was extracted from MEFs using the RNeasy mini kit (Qiagen,74104). Quantitative PCR performed using iScript One-Step RT-PCR Kit with SYBR Green (Bio-Rad, #1708892) on a QuantStudio 5 Real-Time PCR System (Applied Biosystem) using specific primers listed in **Table S1**. Data were normalized to expression of housekeeping genes (β-2-Microglobulin, β-Actin) and fold-change calculated by the $2^{-\Delta\Delta Ct}$ method [1].

Immunoblotting. MEF protein extracts prepared as previously described [2] were analyzed using antibodies listed in Supplementary **Table S2**. Membranes were scanned on an Odyssey Imaging System and quantitation performed using Image J 1.53c software (National Institutes of Health, USA).

RAS•GTP pull-down assays. Serum-starved cells or cells treated as indicated with EGF (100ng/ml), Oligomycin (3µm) Antimycin A (5µM) or CCCP (5µM), were processed as previously described [2] and the RAS•GTP levels were quantified with ImageJ software.

Mitochondrial morphology analysis. MEFs were fixed in 4% paraformaldehyde for 15 min at room temperature and immunostained with anti-TOMM40 as described [2]. Images were acquired using a confocal scanning microscope (Leica SP8, Wetzlar, Germany) with a 63x 1.4NA oil immersion objective. Mitochondrial morphology was analyzed using MicroP [3,4] using the following parameter settings. **Preprocessing parameters**: Denoise Filter: Bilateral; Sigma (distance, intensity): (0.5; 0.05). **Segmentation Parameters:** Local Kernel: disk; Radius Range: 4 : 0.5 : 8; Thresholding Mode: Otsu. **Postprocessing parameters:** Normalized threshold: 1; Intensity threshold: 0.2; Size threshold: 10. The Micro-P algorithm classified mitochondrial morphology into 6 representative subtypes: type 1: small globules – small round shaped and fragmented globules; type 2: swollen globules – large round and irregular-shaped globules; type 3: straight tubules – linear tubules with varied lengths; type 4: twisted tubules – curved tubules with varied lengths; type 5: loops – donuts and horseshoes-shaped tubules; and type 6: branched tubules – branched tubules with varied length. Quantitation of number of mitochondrial structures per cell, percentage of cytoplasmic area occupied by mitochondria and individual mitochondria size was done using Image J 1.53c software.

Flow Cytometry. All analyses were performed using a FACStar flow cytometer (Becton Dickinson). Fluorescent probes purchased from Invitrogen/Molecular Probes included H_2DCFDA (D-399, 5µM, overall ROS), MitoSOX™ (M36008, 5µM, $mO_2{\bullet}^-$) and JC1 (T3168, 3µM, ΔΨm). Antioxidants evaluated involved NAC (Sigma-Aldrich, A7250, 10mM) and the mitochondrial superoxide scavenger MitoTEMPO (Santa Cruz Biotechnology, SC-221945, 100µM). Mitochondrial mass was evaluated using 150nM MitoTracker™ Green (M7514) as described [5]. Determination of dysfunctional mitochondria was performed by double

staining with MitoTracker™ Green and MitoTracker™ Red CMXRos FM (M7512, 150nM) as described [6]. Carbonyl-cyanide m-chlorophenylhydrazone (CCCP Sigma-Aldrich, C2759, 10µM) was used for controls where appropriate.

ATP and cAMP measurements. Cellular ATP content was quantitated with the ATP Determination kit (Molecular Probes, A22066) and Cyclic AMP levels were measured using an ELISA assay (Enzo Life Sciences, ADI-900-066) according to manufacturer's instructions.

Comparison of electron transport (ET) rates using MitoSOX. Cells were stained with mitochondrial superoxide-sensitive MitoSOX™ (5µM Invitrogen, M36008) and the resulting fluorescence intensity signals were monitored by flow cytometry. ET rates were estimated as described [7] from the slope of graphs plotting the kinetics of fluorescence intensity change (0-120 min) after addition of Oligomycin (3µM , Calbiochem, 495455).

SUPPLEMENTARY TABLES

Table S1: Primer pairs used for qRT-PCR

Gene	*Access. no.*	*Forward (5'–3')*	*Reverse (5'–3')*
**PGC1alpha**	NM_008904.2	AGGAAATCCGAGCGGAGCTGA	GCAAGAAGGCGACACATCGAA
**PRC**	NM_001081214.1	ATTCAGAGCTGCTCGTGTCC	GGGCCCCAAAGGGTCAAT
**NRF1**	NM_001164226.1	TCGGGCATTTATCCCAGAGATGCT	TACGAGATGAGCTATACTGTGTGT
**NRF2**	NM_008065.2	GCAATGTGAGAGCAGGTTCA	GTGGCTACACCAGGCTGTTT
**TFAM-1**	NM_009360.4	AGCCAGGTCCAGCTCACTAA	AAACCCAAGAAAGCATGTGG
**SOD1**	NM_011434	GTGATTGGGATTGCGCAGTA	TGGTTTGAGGGTAGCAGATGAGT
**SOD2**	NM_013671	TTAACGCGCAGATCATGCA	GGTGGCGTTGAGATTGTTCA
**SOD3**	NM_011435	CATGCAATCTGCAGGGTACAA	AGAACCAAGCCGGTGATCTG
**CAT**	NM_009804	TGAGAAGCCTAAGAACGCAATTC	CCCTTCGCAGCCATGTG
**TRX**	X77585	CCGCGGGAGACAAGCTT	GGAATGGAAGAAGGGCTTGATC
**PRX1**	NM_011034	GATCCCAAGCGCACCATT	TAATAAAAAGGCCCCTGAAAGAGAT
**Gpx1**	NM_001329527	GAAGAACTTGGGCCATTTGG	TCTCGCCTGGCTCCTGTTT
**Gpx2**	NM_030677	ACCGATCCCAAGCTCATCAT	CAAAGTTCCAGGACACGTCTGA
**Gpx3**	NM_008161	ACAATTGTCCCAGTGTGTGCAT	TGGACCATCCCTGGGTTTC
**GSTa2**	J03958	CGTCCACCTGCTGGAACTTC	GCCTTCAGCAGAGGGAAAGG
**GSTm2**	J04696	GCTCTTACCACGTGCAGCTT	GGCTGGGAAGAGGAAATGGA
**mB2MF1**	NC_000068.7	ATGGGAAGCCGAACATACTG	CAGTCTCAGTGGGGGTGAAT

Table S2: Antibodies used

Name	Supplier	Cat. Nº	Dilution
**MFN1**	Proteintech	137798-1-AP	1:1000
**MFN2**	Proteintech	12186-1-AP	1:1000
**OPA-1**	BD	612607	1:1000
**VDAC**	Cell Signaling	#4661	1:1000
**DRP1**	Cell Signaling	#8570	1:1000
**Fis1**	Proteintech	10956-1-AP	1:1000
**Tubulin**	Sigma	T5293	1:1000
**TOMM40**	Proteintech	18409-1-AP	1:1000
**NDUFS3**	Abcam	ab14711	1:1000
**UQCRC2**	Abcam	ab14745	1:500
**SDHB**	Invitrogen	21A11AE7	1:500
**COXIV**	Cell Signaling	#4850	1:1000
**βF1**	Ref. [8]		1:1000
**Hexokinase I**	Cell Signaling	#2024	1:1000
**Hexokinase II**	Cell Signaling	#2867	1:1000
**HSP60**	Abcam	ab190828	1:1000
**LDHA**	Cell Signaling	#2012	1:1000
**pAMPK**	Cell Signaling	#2535	1:500
**AMPK**	Cell Signaling	#2603	1:500
**Cleaved Caspase 3**	Cell Signaling	#9661	1:500
**pan-Ras**	Millipore	005-516	1:1000
**MEK1**	Cell Signaling	#9124	1:1000
**BRAF**	Cell Signaling	#9434	1:1000
**ERK**	Cell Signaling	#4696	1:2000
**pERK**	Cell Signaling	#9101	1:2000
**AffiniPure Goat Anti-Rabbit IgG (H+L)**	Jackson Inmunoresearch	111-175-144	1:400

SUPPLEMENTARY FIGURES

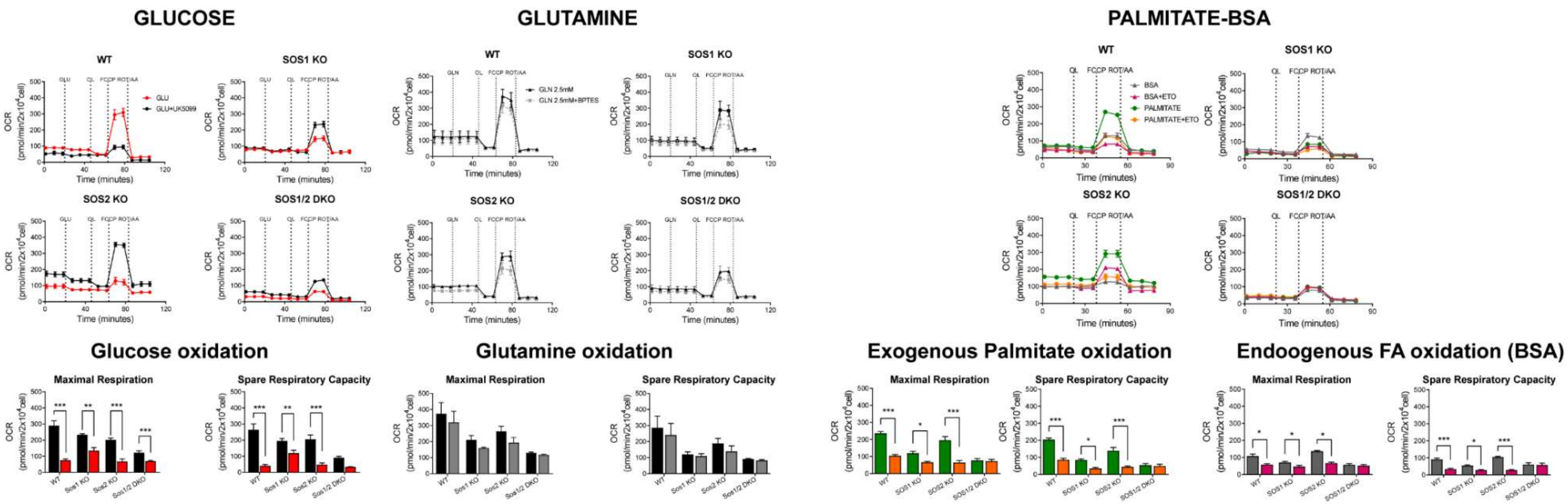

Figure S1. Specific patterns of utilization of oxidation substrates in SOS1-deficient MEFs (Related to Figure 6). Rate of oxidation of different substrate nutrients by WT and SOS-deficient MEFs under conditions of high substrate demand. Seahorse XF Cell Mito Stress Test tracings performed on primary MEFs of the indicated genotypes that had been starved overnight and then treated as described in Materials and Methods under specific conditions designed for subsequent testing of their capability of oxidation of glucose, glutamine, palmitate or endogenous lipids (BSA) as the only available respiratory substrate. OCR was measured under basal conditions or after 1 hour treatment with UK5099 (20µM, 1h) for glucose utilization test, the GSL1 inhibitor BPTES (20µM, 1h) for glutamine utilization tests, or Etomoxir (100µM 1h) for tests of utilization of exogenous fatty acids (Palmitate) or endogenous fatty acids (BSA), followed by the sequential addition of 1.5µM Oligomycin (OL), 1µM FCCP and 1µM Rotenone and Antimycin A (ROT/AA). Bars in the graphs represent the absolute values of Maximal respiration rates and Spare respiratory capacity obtained in the presence or absence of the inhibitors. Results compiled from six independent experiments using at least 5 technical replicates per experiment per genotype. Values expressed as mean ± s.e.m. *** $p < 0.001$; **$p < 0.01$; *, $p < 0.05$. (n=6).

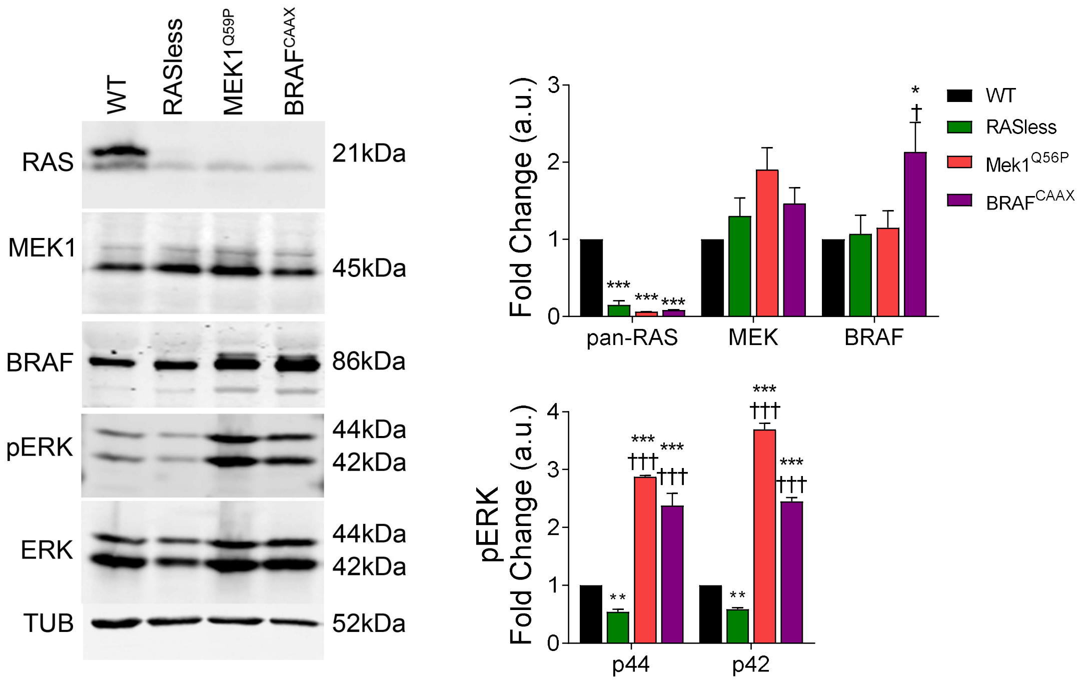

Figure S2. Constitutive activation of the RAF/MEK/ERK pathway in rescued Rasless MEFs. Cell lines of the indicated genotypes (WT, RASless, and Rasless cells infected with retroviral vectors carrying $MEK1^{Q56P}$ or $BRAF^{CAAX}$) were cultured in the presence of 4OHT by 12 days. Representative Western blots (left) and densitometric analysis (right) of the indicated proteins. ERK was used for normalization of pERK, Tubulin (TUB) used as internal loading control. Data expressed as mean ± s.e.m. and compiled from four independent experiments using at least five technical replicates per experiment per genotype. Statistics: * vs WT; † vs RASless; ***, †††$p < 0.001$;** $p < 0.01$; *,† $p < 0.05$. (n=5).

REFERENCES FOR SUPPLEMENTARY MATERIALS

1 Rao X, Huang X, Zhou Z, Lin X. An improvement of the 2ˆ(-delta delta CT) method for quantitative real-time polymerase chain reaction data analysis. *Biostat Bioinforma Biomath* 2013; **3**: 71–85.

2 Liceras-Boillos P, García-Navas R, Ginel-Picardo A, Anta B, Pérez-Andrés M, Lillo C *et al.* Sos1 disruption impairs cellular proliferation and viability through an increase in mitochondrial oxidative stress in primary MEFs. *Oncogene* 2016; **35**: 6389–6402.

3 Peng J-Y, Lin C-C, Chen Y-J, Kao L-S, Liu Y-C, Chou C-C *et al.* Automatic Morphological Subtyping Reveals New Roles of Caspases in Mitochondrial Dynamics. *PLoS Comput Biol* 2011; **7**: e1002212.

4 Valente AJ, Maddalena LA, Robb EL, Moradi F, Stuart JA. A simple ImageJ macro tool for analyzing mitochondrial network morphology in mammalian cell culture. *Acta Histochem* 2017; **119**: 315–326.

5 Monteiro L de B, Davanzo GG, de Aguiar CF, Moraes-Vieira PMM. Using flow cytometry for mitochondrial assays. *MethodsX* 2020; **7**: 100938.

6 Babu D, Leclercq G, Motterlini R, Lefebvre RA. Differential Effects of CORM-2 and CORM-401 in Murine Intestinal Epithelial MODE-K Cells under Oxidative Stress. *Front Pharmacol* 2017; **8**. doi:10.3389/fphar.2017.00031.

7 Maus M, Cuk M, Patel B, Lian J, Ouimet M, Kaufmann U *et al.* Store-Operated Ca 2+ Entry Controls Induction of Lipolysis and the Transcriptional Reprogramming to Lipid Metabolism. *Cell Metab* 2017; **25**: 698–712.

8 Cuezva JM, Krajewska M, De Heredia ML, Krajewski S, Santamaría G, Kim H *et al.* The bioenergetic signature of cancer: A marker of tumor progression. *Cancer Res* 2002; **62**: 6674–6681.
